# Dried Pomegranate Potentiates Anti-Osteoporotic and Anti-Obesity Activities of Red Clover Dry Extracts in Ovariectomized Rats

**DOI:** 10.3390/nu7042622

**Published:** 2015-04-09

**Authors:** Su Jin Kang, Beom Rak Choi, Seung Hee Kim, Hae Yeon Yi, Hye Rim Park, Dong Chul Kim, Seong Hun Choi, Chang Hyun Han, Soo Jin Park, Chang Hyun Song, Sae Kwang Ku, Young Joon Lee

**Affiliations:** 1 The Medical Research Center for Globalization of Herbal Medicine, Daegu Haany University, Gyeongsan, Gyeongsangbuk-Do 712-715, Korea; E-Mails: vegonia1@hanmail.net (S.J.K.); sjp124@gmail.com (S.J.P.); dvmsong@hotmail.com (C.H.S.); 2 Department of Preventive medicine, College of Korean Medicine, Daegu Haany University, 1, Hannydaero, Gyeongsan, Gyeongsangbuk-Do 712-715, Korea; 3 Research Institute, Health-Love Co., Ltd., Anyang, 431-060, Korea; E-Mails: brchoi@health-love.com (B.R.C.); key7413@health-love.com (S.H.K.); Leehaeyun@health-love.com (H.Y.L.); hrpark@health-love.com (H.R.P.); 4 Department of Korean Obstetrics & Gynecology, College of Korean Medicine, Daegu Haany University, Daegu, 704-123, Korea; E-Mail: kdc072@dhu.ac.kr; 5 Department of Histology and Anatomy, College of Korean Medicine, Daegu Haany University, 1, Hannydaero, Gyeongsan, Gyeongsangbuk-Do 712-715, Korea; E-Mail: ck0190@hanmail.net; 6 Department of Medical History & Literature Group, Korea Institute of Oriental Medicine, Daejeon, 305-811, Korea; E-Mail: chhan@kiom.re.kr

**Keywords:** red clover extracts, dried pomegranate concentrate powder, 2:1 mixture, anti-climacteric effects, ovariectomy, rats

## Abstract

Red clover (RC) shows potential activity against menopausal symptoms and pomegranates have antioxidative and beneficial effects on postmenopausal symptoms; thus, we investigated whether the anti-climacteric activity of RC could be enhanced by the addition of dried pomegranate concentrate powder (PCP) extracts in ovariectomized (OVX) rats. Regarding the anti-osteoporotic effects, bone mineral density increased significantly in OVX induced rats treated with 60 and 120 mg/kg of an RC:PCP 2:1 mixture, respectively, compared with OVX control rats. Additionally, femoral, tibia, and L4 bone resorption was decreased in OVX induced control rats treated with the RC:PCP 2:1 mixture (60 and 120 mg/kg), respectively, compared with OVX control rats. Regarding anti-obesity effects, the OVX induced rats treated with 60 and 120 mg/kg of the RC:PCP 2:1 mixture showed a decrease in total fat pad thickness, the mean diameters of adipocytes and the body weights gain compared with OVX induced control rats. The estradiol and bone-specific alkaline phosphatase levels were significantly increased in OVX induced rats treated with the RC:PCP 2:1 mixture (120 mg/kg) compared with OVX induced control rats, also, the uterine atrophy was significantly inhibited in 60 and 120 mg/kg of the RC:PCP 2:1 mixture treatment compared with OVX control rats. In conclusion, our results indicate that PCP enhanced the anti-climacteric effects of RC in OVX rats. The RC:PCP 2:1 mixture used in this study may be a promising new potent and protective agent for relieving climacteric symptoms.

## 1. Introduction

The postmenopausal state is associated with an increased risk of metabolic diseases such as obesity, heart disease, diabetes, and hypertension [1]. These metabolic diseases might be caused directly by estrogen deficiency and may occur partly as secondary effects of obesity due to the orexigenic effects of estrogen deficiency [2]. Estrogen deficiency is considered a common risk factor for developing osteoporosis.

Relief from menopausal symptoms using phytosubstances from plants as an alternative to estrogen/progesterone therapy has gained much attention. Several phytoestrogens (PEs), plant-derived chemicals with antioxidative effects *in vitro*, act as free radical scavengers. Isoflavones, derived from soy and soy derivatives, are the most common PEs and genistein and daidzein are the most abundant and well-studied. This class of PEs can also be found in clover.

Red clover (RC, *Trifolium pratense* L.) botanical dietary supplements have received significant attention for their potential safe use in the treatment of menopausal symptoms, maintenance/improvement of cardiovascular health, and for their reported benign effects on the breast, endometrium, and neural structures [3]. The estrogenic activity of RC is mainly due to isoflavones and, to a lesser extent, coumestans [4]. The isoflavones formononetin, biochanin A, genistein, and daidzein are present in RC as glycosides and malonates [5]. Isoflavones and PEs mimic estrogen [6], and their estrogenic effects in biological systems are believed to be related to their structural similarity to estrogen.

Pomegranates (*Punica granatum* L.) are commonly consumed as fresh fruit, beverages, other food products, dietary supplements, and herbal medicine ingredients [7]. The pomegranate is one of the richest sources of flavonoid antioxidants. The main active substances in pomegranate are polyphenols, which have antioxidative, antimutagenic, anti-inflammatory, and antimicrobial properties [8]. Recent reports showed that pomegranates contain several species of flavonoids and anthocyanidins in their seed oil and juice, and that their antioxidative activities are three times more potent than those of red wine and green tea extract [9,10]. The phytoestrogenic effects of pomegranate [11] are mainly related to the phytoestrogenic effects of isoflavonoids *via* antioxidant and anti-inflammatory pathways [12,13].

Based on the above information, we hypothesized that dried pomegranate concentrate powder (PCP) could potentiate the anti-climacteric effects of RC by increasing the diversity of bioactive isoflavonoids. Thus, we examined whether the anti-climacteric activity of RC could be enhanced by the addition of PCP in ovariectomized (OVX) rats. The anti-climacteric effects of RC, PCP, or RC + PCP were divided into three categories (estrogenic, anti-osteoporotic, and anti-obesity) and evaluated. An estrogen-deficient OVX rat model was used to evaluate the anti-climacteric effects of the mixtures. Since several symptoms of climacterium are clearly induced by ovariectomy within 4 to 6 weeks postoperatively, the rat model was chosen to investigate the mechanisms responsible for menopause-related complications in humans due to many similarities with postmenopausal climacterium symptoms in rats.

## 2. Methods and Materials

### 2.1. Animals and Husbandry

Virgin Sprague-Dawley, specific pathogen-free (SPF) rats (6 weeks old upon receipt) (OrientBio, Seungnam, Korea) were used after acclimatization for 7 days. The animals (4 rats per polycarbonate cage) were kept in a room with a controlled temperature (20–25 °C) and humidity (45%–55%) under 12-h:12-h light:dark cycles. Water and feed (Samyang, Seoul, Korea) were given *ad libitum*. All laboratory animals were treated according to national regulations for the usage and welfare of laboratory animals and approved by the Institutional Animal Care and Use Committee of Daegu Haany University (Gyeongsan, Gyeongbuk, Korea) prior to the experiments. In addition, experiments on osteoporosis were conducted based on United States Food and Drug Administration guidelines [14].

### 2.2. Experimental Groups

The experimental groups were divided into the following 7 groups (8 rats per group): sham control, sham-operated, and vehicle-administered control rats; OVX control, bilateral OVX-operated, and vehicle-administered control rats; RC40, bilateral OVX-operated rats + 40 mg/kg RC; PCP20, bilateral OVX-operated rats + 20 mg/kg PCP; Mix30, bilateral OVX-operated rats + 30 mg/kg RC:PCP 2:1 mixture (g/g); Mix60, bilateral OVX-operated rats + 60 mg/kg RC:PCP 2:1 mixture (g/g); and Mix120, bilateral OVX-operated rats + 120 mg/kg RC:PCP 2:1 mixture (g/g).

### 2.3. Experimental Design

In the present study, virgin female SPF rats (6 weeks old upon receipt) were prepared and a bilateral ovariectomy was performed 7 days after acclimatization. At 28 days after surgery, 8 rats per group were selected based on body weight and RC (40 mg/kg), PCP (20 mg/kg), or an RC:PCP 2:1 mixture (g/g) at 30, 60 and 120 mg/kg of body weight was administered orally by gastric gavage once a day for 84 days. Appropriate amounts of RC, PCP, and the RC:PCP 2:1 mixture (g/g) were directly suspended or dissolved in distilled water and administered in a volume of 5 mL/kg. In OVX and sham control rats, only distilled water was administered orally as a vehicle in equal volumes and periods instead of the herbal formulas. After 84 days of continuous oral administration, the anti-climacteric effects of the treatments were evaluated.

### 2.4. Diets

After acclimatization, the animals were fed a pelleted diet (Superfeed Co., Seoul, Korea) as listed in Table 1 [15].

**Table 1:** Ingredient of diets used in this study.

Ingredient (g/kg Diet)	
Casein	200
l-Cystein	3
Corn starch	150
Sucrose	500
Cellulose	50
Soybean oil	50
Lard	0
Mineral mixture	35
Vitamin mixture	10
Choline bitartrate	2
Energy (kJ/g)	0.88
Protein (% kJ/kg)	13.3
Carbohydrate (% kJ/kg)	47.4
Fat (% kJ/kg)	8.0
Fiber (% kJ/kg)	8.0

### 2.5. Preparation and Administration of the Test Substances

Standardized RC, PCP, and a 2:1 (g/g) mixture were supplied by the sponsor (HEALTH-LOVE Co., Ltd., Anyang, Korea) as a greenish-brown or light pink powder (Supplemental data). The RC preparation contained 8% total isoflavones, 0.62% genistein, 5.43% biochanin A, 3.66% formononetin, and 0.47% daidzein suspended in 24 mg/mL of distilled water. The PCP preparation contained 0.90 mg/g of ellagic acid dissolved in 4 mg/mL of distilled water. The RC:PCP 2:1 mixture (g/g) prepared by the sponsor (7.42% of total isoflavones and 0.29 mg/g of ellagic acid) was also suspended in 24 mg/mL of distilled water. At 28 days after surgery, the RC (40 mg/kg), PCP (20 mg/kg), and RC:PCP 2:1 mixtures (g/g) at 30, 60, and 120 mg/kg of body weight were administered orally by gastric gavage once a day for 84 days. Appropriate amounts of RC, PCP, and the RC:PCP 2:1 mixture (g/g) were directly suspended or dissolved in distilled water and administered in a volume of 5 mL/kg. In OVX and sham control rats, only distilled water was administered orally as a vehicle in equal volumes and periods instead of the herbal formulas.

### 2.6. Menopause Induction by Bilateral Ovariectomy

After acclimatization for 7 days, rats were anesthetized with an intraperitoneal injection of 25 mg/kg of Zoletile (Zoletile 50™; Virbac Laboratories, Carros, France) and maintained with 1%–1.5% isoflurane (Hana Pharmaceutical Co., Hwasung, Korea) in a mixture of 70% N_2_O and 28.5% O_2_. Surgery was performed according to established methods [16]. The second group of rats underwent a sham operation in which a similar incision in the *linea alba* was made but bilateral ovariectomy was not performed.

### 2.7. Body Weight Measurements

Body weight changes were recorded at the time of ovariectomy, 1 day before administration, and once a week from the initiation of administration to termination using an automatic electronic balance (Precisa Instruments, Dietikon, Switzerland) [15]. At ovariectomy, the first administration, and at termination, food, but not water, was withheld from the experimental animals (approximately 18 h prior) to reduce differences due to feeding. In addition, body weight gains were calculated as follows equation:

Equation, Body weight gain (g).

(1) Ovariectomy recovery/induced periods (28 days) = body weight at 1 day before the start of administration (27 days after ovariectomy) − body weight at ovariectomy

(2) After administration (84 days) = body weight at termination − body weight at the start of administration.

### 2.8. Food and Water Consumption Measurements

All rats were placed in individual cages containing 150 g of diet and 250 mL of water and the remaining amounts were measured at 24 h after feed supply using an automatic electronic balance (Precisa Instruments) and a measuring cylinder (Pyrex, Corning, NY, USA), respectively. This was regarded as the individual daily food (g/24 h/rat) and water (mL/24 h/rat) rations [15].

### 2.9. Fecal and Urinary Excretion Measurements

The excreted fecal pellets and urine of individual rats were collected over a 24-h period three times at 28, 49, and 83 days after the first administration of the preparations. The total excreted fecal pellet weight and urine volume were measured using an automatic electronic balance (Precisa Instruments) and a measuring cylinder (Pyrex), respectively [17].

### 2.10. Animal Euthanasia

At 84 days after the first administration, the rats were anesthetized with 50 mg/kg of Zoletile and dissected according to established methods [18].

### 2.11. Serum Biochemistry

For serum biochemical analyses, 10 mL of whole blood was collected from the vena cava at euthanasia and serum separated using a clotting activated serum tube. Serum osteocalcin levels were detected using a Rat Osteocalcin ELISA Kit (Immutopics, San Clemente, CA, USA), and serum bone-specific alkaline phosphatase (bALP) levels were detected using a Rat bALP ELISA Kit (Quidel Corp., San Diego, CA, USA) with an ELISA Reader (Tecan, Männedorf, Switzerland) as described previously [19]. In addition, the serum estradiol content was measured using a chemiluminescent immunoassay (ECLIA, Modular E 170C, Estradiol II 03000079 122; Roche Diagnostics, Penzberg, Germany).

### 2.12. Measurement of the Bone Mineral Density (BMD) and Failure Load (FL)

The total, epiphyseal, and mid-shaft BMD of the right femur, tibia, and L5 region were determined using dual-energy X-ray absorptionmetry (Norland pDEXA bone densitometer; Fort Atkinson, WI, USA). In addition, bone strength was detected as the FL. The FL of the right femur and tibia mid-shaft regions was determined using a three-point bending test to failure using a computerized testing machine (SV-H1000; Japan Instrumentation System Co., Nara, Japan) as Newton (*N*) [19].

### 2.13. Histological Procedures

Samples were fixed in 10% neutral buffered formalin (NBF). After paraffin embedding, 3–4-μm serial sections were prepared and then stained with hematoxylin and eosin. The total thickness of the dorsal abdominal fat pads (in mm/rat) were measured using an automated image analysis process (*i*Solution FL version 9.1; IMT *i*-Solution Inc., Quebec, Canada), and the mean diameters of the dorsal abdominal white adipocytes (in μm) were calculated in restricted view fields (at least ten white adipocytes per fat pad according to established methods [18]). In addition, the total (mm/uterus), mucosal (μm/uterus), and epithelial (μm/uterus) thicknesses of the uterus were determined along with the percentage of uterine glands located in the mucosa (%/uterine mucosa).

The left sides of the femur and tibia with L4 in each rat were separated and fixed in 10% NBF, then decalcified in decalcifying solution (24.4% formic acid and 0.5 N sodium hydroxide) for 5 days, embedded in paraffin, and stained with Safranin O. Histomorphometric measurements of bone mass and structure with bone resorption in a uniform area of epiphyseal or cortical bone in the femur, tibia, or L4 were conducted using an automated image analyzer under microscopy (Nikon, Tokyo, Japan). The trabecular bone volume (TV/BV, TBV; %), trabecular bone thickness (μm/trabecular bone), number (mean number of trabecular bone/epiphyseal regions), length (mm/trabecular bone), and cortical bone thickness (μm/mid-shaft cortical bone) were measured for bone mass and structure, and the osteoclast cell number (mean osteoclast cell number/epiphyseal region) and ratio (OS/BS; %) were measured for bone resorption as described previously [16].

### 2.14. Statistical Analyses

The data are presented as means ± standard deviation. Significant differences among means within a group were examined using a parametric method (one-way ANOVA) or non-parametric method (Kruskal-Wallis H test). Variance homogeneity was evaluated using the Levene test. If the Levene test indicated no significant deviation from variance homogeneity, the obtained data were analyzed with a one-way ANOVA followed by the Tukey test as a post hoc test. In cases where significant deviations from variance homogeneity were observed, the data were analyzed using the Kruskal-Wallis H test followed by Tamhane post hoc test. The analyses were conducted using SPSS for Windows (Release 14K; SPSS Inc., Chicago, IL, USA).

## 3. Results

### 3.1. Effects on Body Weight and Gains

**Figure 1:**
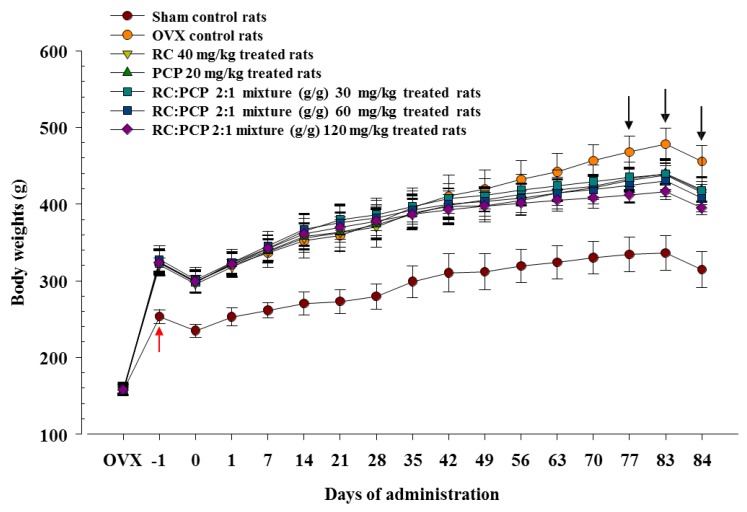
Significant (*p <* 0.01) increases of body weights were detected in all ovariectomized (OVX) rats as compared with sham control rats in this experiment (red arrow). However, significant (*p <* 0.01 or *p <* 0.05) decreases of body weights were demonstrated in mixture 120 mg/kg treated rats from 56 days after initial administration, from 70 days after initial treatment in red clover (RC), pomegranate concentrate powder (PCP), mixture 60 mg/kg and from 77 days after initial treatment in all treated group as compared with OVX control rats, respectively (arrows). Values are expressed mean ± S.D. of eight rats.

For 4 weeks, the body weights of the OVX rats increased more than the body weights of the sham controls. However, the increases in the OVX rats were significantly attenuated by RC and PCP in the 30, 60, and 120 mg/kg mixtures. A significant decrease in body weight was observed in the 120 mg/kg RC:PCP 2:1 mixture (g/g)-treated rats at 56 and 63 days after initial administration and 70 days after initial treatment in the 40 mg/kg RC-, 20 mg/kg PCP-, and 60 mg/kg RC:PCP 2:1 mixture (g/g)-treated rats as compared with the OVX control rats. In addition, all of the test substance-treated OVX rats showed significant decreases in body weight gain compared with the OVX control rats during the 84-day administration period. Specifically, rats treated with the 120 mg/kg mixture showed dramatic decreases in body weight compared with the single formula RC and PCP-treated rats at 77 days after administration (Figure 1).

### 3.2. Effects on Food and Water Consumption

The OVX control rats showed significant increases in food and water consumption compared with the sham control rats. No meaningful changes in daily food and water consumption were observed in the RC-, PCP-, and 30, 60, and 120 mg/kg mixture-treated rats compared with the OVX control rats. In addition, the food and water consumption of the rats was unchanged for all three dosages of the mixture when compared with the single formula of RC and PCP at 28, 49, and 83 days after initial administration (Table 2 and Table 3).

**Table 2:** Food consumptions in OVX rats.

Groups	Food Consumption (g/24 h/Rat): Days after Initial Treatment		
	28	49	83
Controls			
Sham	15.55 ± 1.42	14.19 ± 1.70	12.47 ± 1.56
OVX	20.29 ± 2.61 ^b^	18.80 ± 2.61 ^b^	17.13 ± 1.62 ^b^
RC 40 mg/kg	19.16 ± 3.54	18.93 ± 4.06 ^b^	16.89 ± 2.82 ^b^
PCP 20 mg/kg	18.98 ± 3.06	17.47 ± 2.07	16.96 ± 2.40 ^b^
RC:PCP 2:1 mixture (g/g)			
30 mg/kg	18.93 ± 1.83	19.35 ± 2.12 ^b^	17.00 ± 2.54 ^b^
60 mg/kg	19.65 ± 3.50	19.92 ± 3.74 ^a^	17.92 ± 4.40 ^a^
120 mg/kg	18.49 ± 1.94	17.12 ± 2.97	16.83 ± 2.30 ^b^

Values are expressed mean ± S.D. of eight rats. ^a^ *p <* 0.01 and ^b^ *p <* 0.05 as compared with sham control by Tukey test; OVX = Bilateral ovariectomy; PCP = Pomegranate Concentrate Powder; RC = Red clover dry extracts.

**Table 3:** Water consumptions in OVX rats.

Groups	Water Consumption (mL/24 h/Rat): Days after Initial Treatment		
	28	49	83
Controls			
Sham	26.75 ± 3.49	37.00 ± 3.55	32.25 ± 4.77
OVX	33.38 ± 5.13	48.88 ± 5.25 ^c^	45.25 ± 5.97
RC 40 mg/kg	34.75 ± 7.25	46.13 ± 4.73 ^d^	44.75 ± 12.34
PCP 20 mg/kg	36.13 ± 7.70	47.25 ± 10.43	52.00 ± 13.91 ^a^
RC:PCP 2:1 mixture (g/g)			
30 mg/kg	33.00 ± 6.16	52.13 ± 14.47	48.63 ± 9.83 ^b^
60 mg/kg	38.38 ± 8.60 ^b^	51.13 ± 8.82^d^	50.50 ± 9.30 ^a^
120 mg/kg	35.38 ± 4.78	46.00 ± 4.54^d^	41.38 ± 5.95

Values are expressed mean ± S.D. of eight rats; ^a^ *p <* 0.01 and ^b^ *p <* 0.05 as compared with sham control by Tukey test; ^c^ *p <* 0.01 and ^d^ *p <* 0.05 as compared with sham control by Tamhane test; OVX = Bilateral ovariectomy; PCP = Pomegranate Concentrate Powder; RC = Red clover dry extracts.

### 3.3. Effects on Urine Volume and Fecal Excretion

Urinary and fecal excretion in the OVX control rats was unchanged compared with that in the sham control rats. The 30, 60, and 120 mg/kg mixtures increased urinary excretion significantly compared with the OVX controls from 49 days after initial administration. Notably, urinary excretion in the 60 and 120 mg/kg mixture-treated rats and fecal excretion in the 120 mg/kg mixture treated rats increased more than in rats treated with a single formula of RC and PCP at 83 days after administration (Table 4 and Table 5).

**Table 4:** Urine volumes in OVX rats.

Groups	Urine Volume (mL/24 h/Rat): Days after Initial Treatment		
	28	49	83
Controls			
Sham	2.33 ± 0.81	2.88 ± 0.50	3.65 ± 1.00
OVX	2.36 ± 0.75	3.00 ± 0.50	4.09 ± 0.77
RC 40 mg/kg	2.50 ± 0.90	4.60 ± 1.06 ^ac^	5.59 ± 0.77
PCP 20 mg/kg	2.33 ± 0.71	4.00 ± 0.98	5.49 ± 0.81
RC:PCP 2:1 mixture (g/g)			
30 mg/kg	2.58 ± 1.08	4.60 ± 0.86 ^ac^	6.28 ± 1.41 ^ad^
60 mg/kg	3.90 ± 1.02 ^bdfh^	5.46 ± 0.60 ^ach^	8.80 ± 1.69 ^aceg^
120 mg/kg	4.28 ± 0.93 ^aceg^	6.48 ± 1.10 ^aceg^	10.75 ± 1.88 ^aceg^

Values are expressed mean ± S.D. of eight rats; ^a^ *p <* 0.01 and ^b^ *p <* 0.05 as compared with sham control by Tukey test; ^c^ *p <* 0.01 and ^d^ *p <* 0.05 as compared with OVX control by Tukey test; ^e^ *p <* 0.01 and ^f^ *p <* 0.05 as compared with RC 40 mg/kg treated rats by Tukey test; ^g^ *p <* 0.01 and ^h^ *p <* 0.05 as compared with PCP 20 mg/kg treated rats by Tukey test; OVX = Bilateral ovariectomy; PCP = Pomegranate Concentrate Powder; RC = Red clover dry extracts.

**Table 5:** Fecal excretions in OVX rats.

Groups	Fecal Excretion (g/24 h/Rat): Days after Initial Treatment		
	28	49	83
Controls			
Sham	8.80 ± 2.53	8.23 ± 2.12	9.61 ± 3.33
OVX	9.97 ± 3.13	10.44 ± 4.14	12.09 ± 2.47
RC 40 mg/kg	12.81 ± 4.31	14.91 ± 3.07 ^a^	16.43 ± 1.73 ^il^
PCP 20 mg/kg	10.76 ± 4.50	14.58 ± 2.76 ^a^	16.02 ± 1.03 ^jl^
RC:PCP 2:1 mixture (g/g)			
30 mg/kg	10.00 ± 0.90	15.00 ± 2.77 ^a^	16.19 ± 2.51 ^j^
60 mg/kg	13.33 ± 2.04 ^j^	18.54 ± 2.05 ^ac^	18.98 ± 1.64 ^ikp^
120 mg/kg	12.48 ± 4.38	19.86 ± 3.31 ^acfh^	19.41 ± 1.33 ^ikno^

Values are expressed mean ± S.D. of eight rats; ^a^ *p <* 0.01 and ^b^ *p <* 0.05 as compared with sham control by Tukey test; ^c^ *p <* 0.01 and ^d^ *p <* 0.05 as compared with OVX control by Tukey test; ^e^ *p <* 0.01 and ^f^ *p <* 0.05 as compared with RC 40 mg/kg treated rats by Tukey test; ^g^ *p <* 0.01 and ^h^ *p <* 0.05 as compared with PCP 20 mg/kg treated rats by Tukey test; ^i^ *p <* 0.01 and ^j^ *p <* 0.05 as compared with sham control by Tamhane test; ^k^ *p <* 0.01 and ^l^ *p <* 0.05 as compared with OVX control by Tamhane test; ^m^ *p <* 0.01 and ^n^ *p <* 0.05 as compared with RC 40 mg/kg treated rats by Tamhane test; ^o^ *p <* 0.01 and ^p^ *p <* 0.05 as compared with PCP 20 mg/kg treated rats by Tamhane test; OVX = Bilateral ovariectomy; PCP = Pomegranate Concentrate Powder; RC = Red clover dry extracts.

### 3.4. Effects on Serum Biochemistry

**Figure 2:**
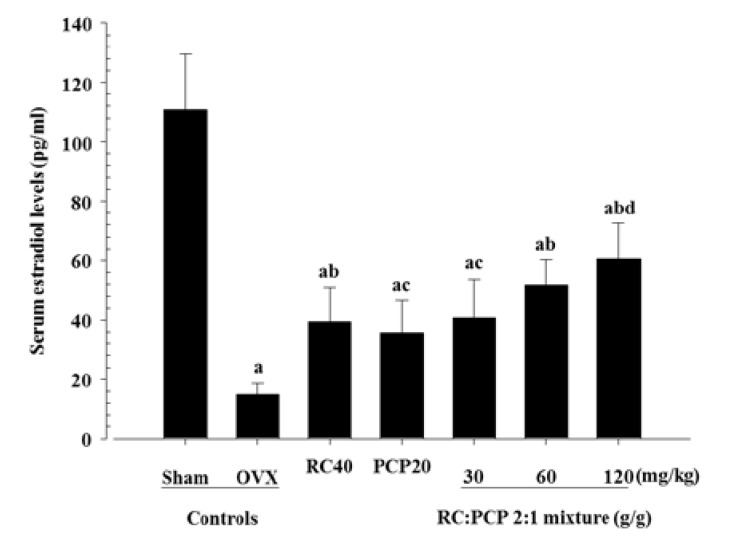
Significant changes of the serum estradiol levels were demonstrated in mixture 120 mg/kg treated rats as compared with those of single formula of PCP treated rats, respectively. Values are expressed mean ± S.D. of eight rats. ^a^ *p <* 0.01 as compared with sham control; ^b^ *p <* 0.01 and ^c^ *p <* 0.05 as compared with OVX control; ^d^ *p <* 0.05 as compared with PCP treated rats by Tamhane test.

**Figure 3:**
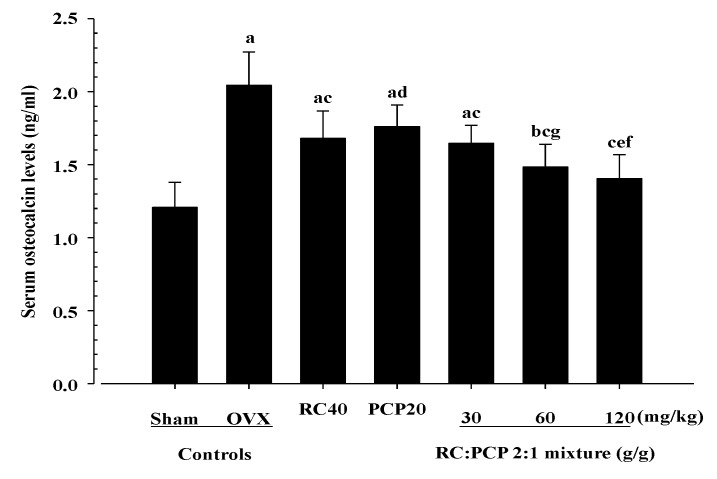
Significant changes of the serum osteocalcin levels were demonstrated in mixture 60 and 120 mg/kg treated rats as compared with those of single formula of RC or/and PCP treated rats, respectively. Values are expressed mean ± S.D. of eight rats. ^a^ *p <* 0.01 and ^b^ *p <* 0.05 as compared with sham control; ^c^ *p <* 0.01 and ^d^ *p <* 0.05 as compared with OVX control; ^e^ *p <* 0.05 as compared with RC treated rats; ^f^ *p <* 0.01 and ^g^ *p <* 0.05 as compared with PCP treated rats by Tukey test.

**Figure 4:**
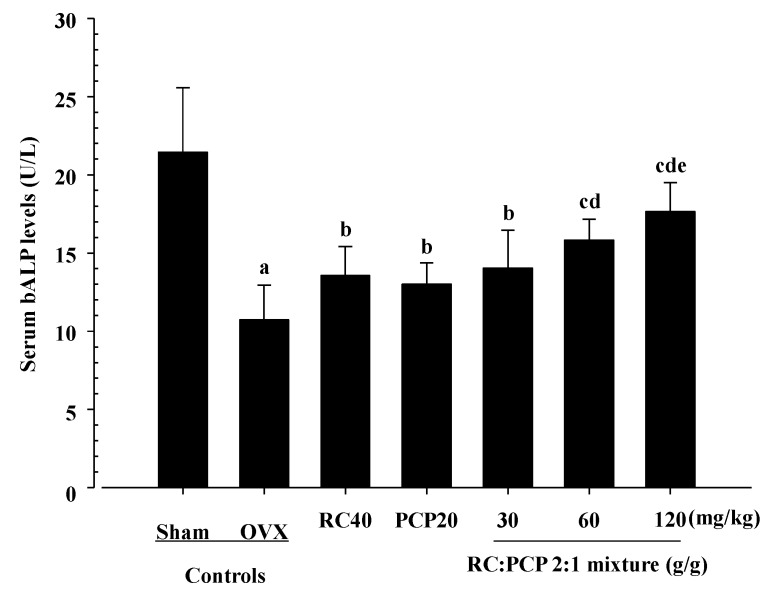
Significant changes of the serum bALP levels were demonstrated in mixture 60 and 120 mg/kg treated rats as compared with those of single formula of RC or/and PCP treated rats, respectively. Values are expressed mean ± S.D. of eight rats. ^a^ *p <* 0.01 and ^b^ *p <* 0.05 as compared with sham control; ^c^ *p <* 0.01 as compared with OVX control; ^d^ *p <* 0.05 as compared with RC treated rats; ^e^ *p <* 0.01 and ^f^ *p <* 0.05 as compared with PCP treated rats by Tamhane test.

Serum estradiol and serum bALP levels were decreased in the OVX control rats and increased in the RC-, PCP-, and 30, 60, and 120 mg/kg mixture-treated rats. In addition, serum osteocalcin was increased in the OVX control rats but decreased in all of the test formula-treated rats. Specifically, the 120 mg/kg mixture-treated rats showed significant increases in serum estradiol content compared with rats treated with a single formula of PCP (Figure 2). In particular, the 120 mg/kg mixture-treated rats showed significant decreases in serum osteocalcin (Figure 3) and increases in serum bALP compared with rats treated with a single formula of RC and PCP (Figure 4).

### 3.5. Effects on BMD and FL

**Figure 5:**
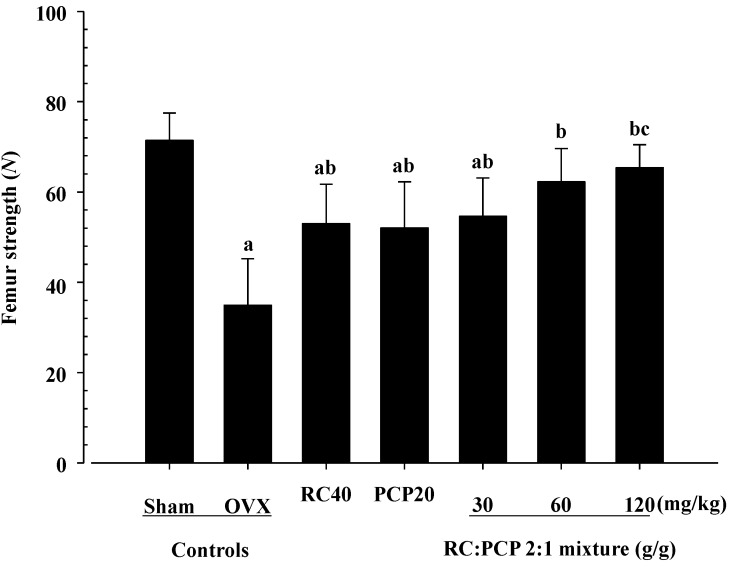
The strengths of femur mid-shaft regions in mixture 120 mg/kg treated rats were significantly decreased as compared with those of single formula of PCP treated rats, respectively. Values are expressed mean ± S.D. of eight rats. ^a^ *p <* 0.01 as compared with sham control; ^b^ *p <* 0.01 as compared with OVX control; ^c^ *p <* 0.05 as compared with PCP 20 mg/kg treated rats by Tukey test.

**Figure 6:**
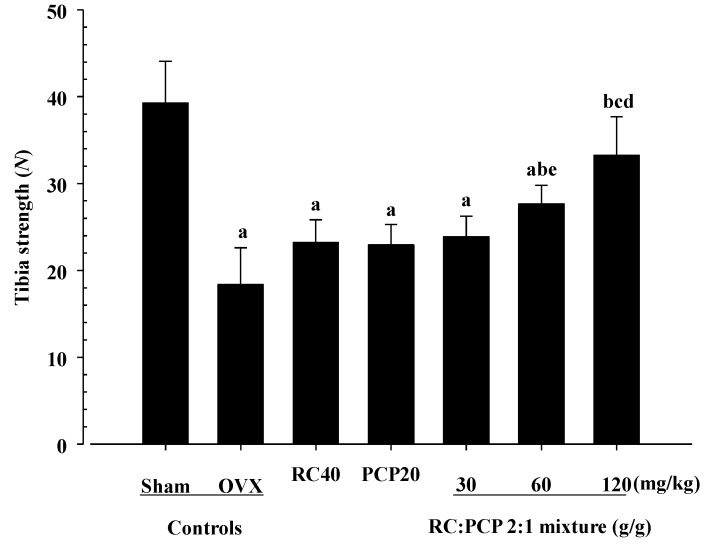
The strengths of tibia mid-shaft regions in mixture 60 and 120 mg/kg treated rats were significantly decreased as compared with those of single formula of RC or/and PCP treated rats, respectively. Values are expressed mean ± S.D. of eight rats. ^a^ *p <* 0.01 as compared with sham control; ^b^ *p <* 0.01 as compared with OVX control; ^c^ *p <* 0.01 as compared with RC 40 mg/kg treated rats; ^d^ *p <* 0.01 and ^e^ *p <* 0.05 as compared with PCP 20 mg/kg treated rats by Tamhane test.

The total, epiphyseal, and mid-shaft BMDs of the femur and tibia and L5 region in the OVX control rats were decreased compared with those in the sham controls. However, 60 and 120 mg/kg mixture-treated OVX rats showed significant increases in BMD compared with the OVX control rats. Compared with the sham control rats, the OVX control rats showed a decrease in strength of the femur and tibia mid-shaft regions. In contrast, the FL of the femur was significantly increased in all test substance-administered rats compared with the OVX rats. The FL of the tibia was also significantly increased in 60 and 120 mg/kg mixture treated-rats compared with the OVX rats. Specifically, the 120 mg/kg mixture-treated rats showed noticeable increases in BMD and in the FL of the tibia compared with rats treated with a single formula of RC and PCP (Table 6 and Figure 5 and Figure 6).

### 3.6. Histopathology

**Figure 7:**
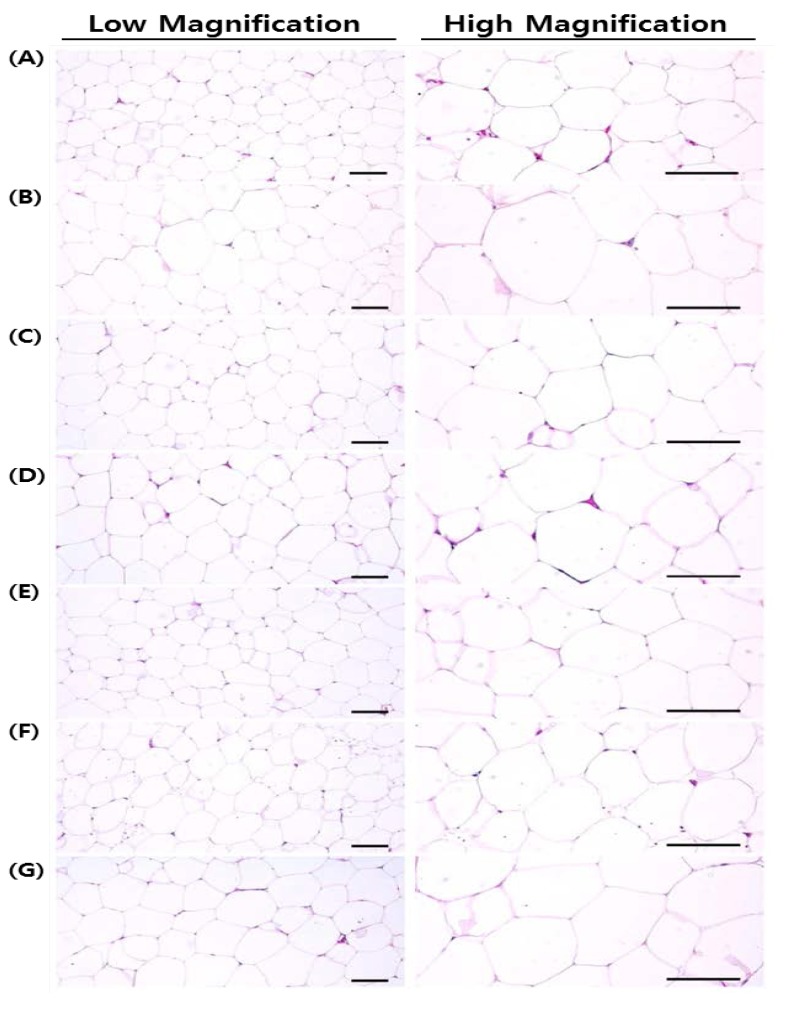
Representative histological images of the adipocytes, taken from sham-operated or OVX rats abdominal fat pads deposited into left dorsal abdominal muscles. Obvious increases of mean adipocyte diameters, adipocyte hypertrophic changes, were demonstrated in OVX control rats, due to noticeable deposition of adipose tissues on the abdominal cavity. However, noticeable decreases of the mean diameters of adipocytes were detected in all test substance administrated rats as compared with OVX control rats, respectively. Especially, mixture 120 and 60 mg/kg treated rats also showed dramatic inhibitory activities on the hypertrophy of adipocytes in the abdominal cavity as compared with those of single formula of RC and PCP treated rats, respectively. (**A**); Shame control rat, (**B**); OVX control rat, (**C**); RC 40 mg/kg treated rat, (**D**); PCP 20 mg/kg treated rat, (**E**); RC:PCP 2:1 mixture (g/g) 120 mg/kg treated rat, (**F**); RC:PCP 2:1 mixture (g/g) 60 mg/kg treated rat, (**G**); RC:PCP 2:1 mixture (g/g) 30 mg/kg treated rat. All H&E stained. Scale bars = 240 μm.

**Figure 8:**
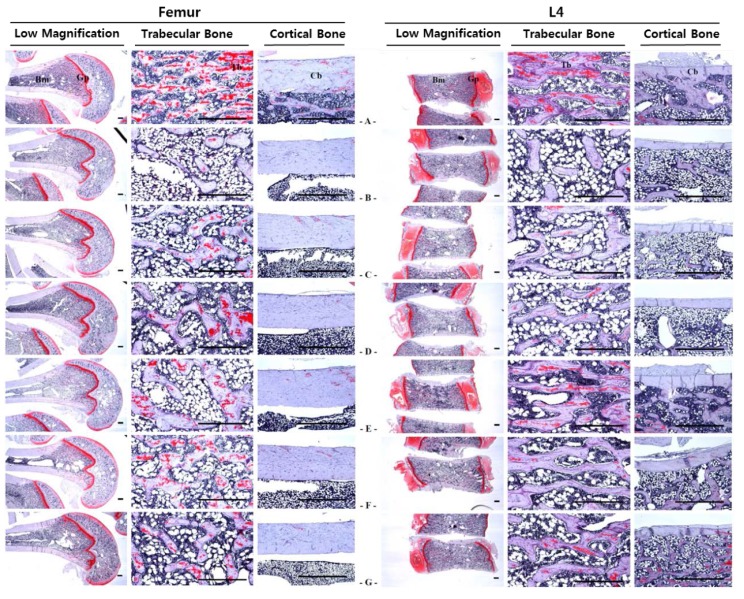
Representative histological profiles of the femur and L4, taken from sham-operated or OVX rats. Although relatively well-developed trabecular and cortical bone were observed in the long bone-femur and the short bone-L4 of sham control rats, classical osteoporotic histological profiles were demonstrated in OVX control rats as dramatic decreases of trabecular and cortical bone masses, increase of connective tissues in periosteum of cortical bone results from resorption of osteoid tissues related to osteocalst activations in this study. However, dramatic increases of the bone mass and structures, the both trabecular and cortical bones were detected in all test substance administered rats including PCP as compared with OVX control rats, related to their inhibitory activities on osteoclast cell activities, respectively. Especially, mixture 120 and 60 mg/kg treated rats also showed noticeable inhibition of the bone losses and osteoclast cell activations as compared with those of single formula of RC and PCP treated rats, respectively. (**A**); Shame control rat, (**B**); OVX control rat, (**C**); RC 40 mg/kg treated rat, (**D**); PCP 20 mg/kg treated rat, (**E**); RC:PCP 2:1 mixture (g/g) 120 mg/kg treated rat, (**F**); RC:PCP 2:1 mixture (g/g) 60 mg/kg treated rat, (**G**); RC:PCP 2:1 mixture (g/g) 30 mg/kg treated rat. L4; Fourth lumbar vertebrae, Cb; cortical bone, Tb; trabecular bone, Bm; bone marrow, Gp; growth plate. All Safranin O stained. Scale bars = 480 μm.

Compared with the OVX control rats, the test substance-treated rats showed significant decreases in abdominal fat pad thickness and mean adipocyte diameter. Notably, when compared with the single formula RC and PCP-treated rats, the 60 and 120 mg/kg mixtures inhibited adipose tissue deposition and adipocyte hypertrophy noticeably in the abdominal cavity (Table 7 and Figure 7). Also, the 60 and 120 mg/kg mixture-treated rats showed significant increment of ovariectomy-induced uterine atrophic histopathological changes compared with rats treated with a single formula of RC and PCP (Table 7). Bone loss and osteoclast activation were inhibited to a greater extent in the 120 mg/kg mixture-treated rats than in the single formula RC and PCP-treated rats (Table 8, Table 9, Table 10 and Figure 8).

**Table 6:** Bone mineral density of right femur and tibia with L5 in OVX rats.

Groups	Control		References		RC:PCP 2:1 Mixture (g/g)		
	Sham	OVX	RC 40 mg/kg	PCP 20 mg/kg	30 mg/kg	60 mg/kg	120 mg/kg
Femur							
Total	0.149 ± 0.010	0.121 ± 0.006 ^a^	0.128 ± 0.004 ^a^	0.128 ± 0.005 ^a^	0.130 ± 0.004 ^a^	0.135 ± 0.004 ^ac^	0.139 ± 0.005 ^bcfg^
Neck	0.158 ± 0.012	0.126 ± 0.003 ^i^	0.138 ± 0.010	0.134 ± 0.004 ^jl^	0.140 ± 0.011	0.147 ± 0.004 ^ko^	0.154 ± 0.008 ^ko^
Mid-shaft	0.126 ± 0.005	0.106 ± 0.004 ^a^	0.114 ± 0.003 ^ac^	0.112 ± 0.004 ^a^	0.114 ± 0.005 ^ac^	0.120 ± 0.003 ^acg^	0.123 ± 0.003 ^ceg^
Tibia							
Total	0.134 ± 0.010	0.104 ± 0.003 ^a^	0.113 ± 0.007 ^ad^	0.111 ± 0.003 ^a^	0.113 ± 0.005 ^ad^	0.121 ± 0.005 ^ach^	0.124 ± 0.004 ^aceg^
Neck	0.146 ± 0.007	0.116 ± 0.006 ^a^	0.126 ± 0.005 ^a^	0.124 ± 0.004 ^a^	0.128 ± 0.014 ^a^	0.134 ± 0.006 ^bc^	0.138 ± 0.005 ^cfg^
Mid-shaft	0.119 ± 0.012	0.085 ± 0.007 ^i^	0.096 ± 0.005 ^j^	0.095 ± 0.005 ^i^	0.097 ± 0.008 ^j^	0.102 ± 0.004 ^k^	0.111 ± 0.008 ^kno^
L4-Total	0.139 ± 0.009	0.109 ± 0.005 ^a^	0.120 ± 0.005 ^a^	0.118 ± 0.006 ^a^	0.120 ± 0.010 ^ad^	0.126 ± 0.004 ^ac^	0.130 ± 0.008 ^ch^

Values are expressed mean ± S.D. of eight rats, g/cm^2^ of bone; ^a^ *p <* 0.01 and ^b^ *p <* 0.05 as compared with sham control by Tukey test; ^c^ *p <* 0.01 and ^d^ *p <* 0.05 as compared with OVX control by Tukey test; ^e^ *p <* 0.01 and ^f^ *p <* 0.05 as compared with RC 40 mg/kg treated rats by Tukey test; ^g^ *p <* 0.01 and ^h^ *p <* 0.05 as compared with PCP 20 mg/kg treated rats by Tukey test; ^i^ *p <* 0.01 and ^j^ *p <* 0.05 as compared with sham control by Tamhane test; ^k^ *p <* 0.01 and ^l^ *p <* 0.05 as compared with OVX control by Tamhane test; ^m^ *p <* 0.01 and ^n^ *p <* 0.05 as compared with RC 40 mg/kg treated rats by Tamhane test; ^o^ *p <* 0.01 and ^p^ *p <* 0.05 as compared with PCP 20 mg/kg treated rats by Tamhane test; OVX = Bilateral ovariectomy; PCP = Pomegranate Concentrate Powder; RC = Red clover dry extracts; L4 = fourth lumbar vertebrae.

**Table 7:** Histopathology-histomorphometry for abodminal fat pads and uterus in OVX rats.

Groups	Control		References		RC:PCP 2:1 Mixture (g/g)		
	Sham	OVX	RC 40 mg/kg	PCP 20 mg/kg	30 mg/kg	60 mg/kg	120 mg/kg
Fat pads							
Total Th (mm)	4.43 ± 0.92	9.85 ± 1.60 ^a^	7.70 ± 1.01 ^ac^	8.02 ± 0.54 ^ac^	7.62 ± 1.18 ^ac^	6.37 ± 0.57 ^acg^	5.55 ± 0.71 ^cdf^
Adipocyte DM (μm)	82.20 ± 12.37	158.17 ± 19.42 ^a^	117.50 ± 14.07 ^ac^	127.82 ± 15.14 ^ac^	116.00 ± 14.21 ^ac^	101.74 ± 11.48 ^cf^	99.03 ± 10.07 ^cf^
Uterus							
Total Th (mm)	3.21 ± 0.65	0.54 ± 0.11 ^h^	0.89 ± 0.10 ^hj^	0.73 ± 0.09 ^hk^	0.84 ± 0.15 ^hk^	1.10 ± 0.10 ^hjmn^	1.45 ± 0.36 ^hjo^
Epi Th (μm)	38.47 ± 5.18	9.76 ± 2.26 ^a^	16.90 ± 1.51 ^ac^	19.16 ± 1.72 ^ac^	18.79 ± 2.93 ^ac^	22.81 ± 2.02 ^acd^	24.28 ± 2.31 ^acdg^
Mucosa Th (μm)	967.28 ± 220.32	176.98 ± 21.58 ^h^	227.65 ± 31.82 ^hj^	261.87 ± 32.06 ^hj^	286.24 ± 41.40 ^hj^	420.77 ± 94.19 ^hjo^	484.97 ± 56.84 ^hjln^
UG percentage (%)	28.78 ± 4.83	4.85 ± 2.17 ^a^	9.28 ± 1.53 ^ac^	11.03 ± 3.19 ^ac^	11.34 ± 3.50 ^ac^	17.67 ± 2.71 ^acdf^	20.10 ± 3.13 ^acdf^

Values are expressed mean ± S.D. of eight rats; ^a^ *p <* 0.01 and ^b^ *p <* 0.05 as compared with sham control by Tukey test; ^c^ *p <* 0.01 as compared with OVX control by Tukey test; ^d^ *p <* 0.01 and ^e^ *p <* 0.05 as compared with RC 40 mg/kg treated rats by Tukey test; ^f^ *p <* 0.01 and ^g^ *p <* 0.05 as compared with PCP 20 mg/kg treated rats by Tukey test; ^h^ *p <* 0.01 and ^i^ *p <* 0.05as compared with sham control by Tamhane test; ^j^ *p <* 0.01 and ^k^ *p <* 0.05 as compared with OVX control by Tamhane test; ^l^ *p <* 0.01 and ^m^ *p <* 0.05 as compared with RC 40 mg/kg treated rats by Tamhane test; ^n^ *p <* 0.01 and ^o^ *p <* 0.05 as compared with PCP 20 mg/kg treated rats by Tamhane test; OVX = Bilateral ovariectomy; PCP = Pomegranate Concentrate Powder; RC = Red clover dry extracts; Th = thickness; DM = diameter; Epi = epithelium; UG = uterine gland; FC = fatty change.

**Table 8:** Histopathology-histomorphometry for bone mass and resorption of left femur in OVX rats.

Groups	Control		References		RC:PCP 2:1 Mixture (g/g)		
	Sham	OVX	RC 40 mg/kg	PCP 20 mg/kg	30 mg/kg	60 mg/kg	120 mg/kg
Bone mass and structure							
TBV, BV/TV	47.27 ± 11.66	15.88 ± 2.97 ^i^	22.83 ± 2.96 ^ik^	19.20 ± 1.70 ^i^	23.48 ± 3.25 ^jk^	28.94 ± 2.85 ^kno^	33.52 ± 6.51 ^kno^
Tbn	23.00 ± 3.07	11.38 ± 1.92 ^a^	16.00 ± 2.27 ^ac^	15.63 ± 1.69 ^ac^	16.75 ± 1.83 ^ac^	18.38 ± 1.30 ^ac^	20.50 ± 0.93 ^ceg^
Tbl	6.81 ± 1.68	1.99 ± 0.21 ^i^	3.42 ± 0.49 ^jk^	3.08 ± 0.90 ^i^	3.56 ± 1.12 ^j^	5.08 ± 0.25 ^kmo^	5.78 ± 0.42 ^kmo^
Tbt	144.33 ± 11.68	90.54 ± 15.24 ^a^	112.08 ± 11.01 ^ac^	104.83 ± 7.23 ^a^	112.30 ± 10.00 ^ac^	124.82 ± 6.89 ^acg^	133.80 ± 8.50 ^ceg^
Cbt-shaft	848.67 ± 91.46	630.65 ± 10316 ^j^	737.86 ± 49.96	722.33 ± 20.24	741.84 ± 50.86	786.77 ± 22.00^o^	805.56 ± 37.42 ^lo^
Bone resorption							
Ocn	8.25 ± 2.38	31.38 ± 6.02 ^i^	19.75 ± 2.31 ^il^	21.88 ± 3.00 ^il^	19.63 ± 3.20 ^il^	14.88 ± 2.23 ^ikno^	12.50 ± 2.62 ^kmo^
OS/BS	4.28 ± 1.58	14.09 ± 1.96 ^a^	11.00 ± 0.95 ^ac^	12.62 ± 1.69 ^a^	10.83 ± 1.32 ^ac^	9.04 ± 1.35 ^acg^	7.78 ± 1.32 ^aceg^

Values are expressed mean ± S.D. of eight rats; ^a^ *p <* 0.01 and ^b^ *p <* 0.05 as compared with sham control by Tukey test; ^c^ *p <* 0.01 and ^d^ *p <* 0.05 as compared with OVX control by Tukey test; ^e^ *p <* 0.01 and ^f^ *p <* 0.05 as compared with RC 40 mg/kg treated rats by Tukey test; ^g^ *p <* 0.01 and ^h^ *p <* 0.05 as compared with PCP 20 mg/kg treated rats by Tukey test; ^i^ *p <* 0.01 and ^j^ *p <* 0.05 as compared with sham control by Tamhane test; ^k^ *p <* 0.01 and ^l^ *p <* 0.05 as compared with OVX control by Tamhane test; ^m^ *p <* 0.01 and ^n^ *p <* 0.05 as compared with RC 40 mg/kg treated rats by Tamhane test; ^o^ *p <* 0.01 and ^p^ *p <* 0.05 as compared with PCP 20 mg/kg treated rats by Tamhane test; OVX = Bilateral ovariectomy; PCP = Pomegranate Concentrate Powder; RC = Red clover dry extracts; L4 = fourth lumbar vertebrae; Cbt = Cortical bone thickness (Cross thickness; μm); Tbl = Trabecular bone length (Longitudinal thickness; mm); Tbn = Trabecular bone number (N/epiphyseal); Tbt = Trabecular bone thickness (Cross thickness; μm); TV/BV = Trabecular bone volume (%); OS/BS = Osteoclast cell surface/bone surface (%); Ocn = Osteoclast cell number (N/epiphyseal).

**Table 9:** Histopathology-histomorphometry for bone mass and resorption of tibia in OVX rats.

Groups	Control		References		RC:PCP 2:1 Mixture (g/g)		
	Sham	OVX	RC 40 mg/kg	PCP 20 mg/kg	30 mg/kg	60 mg/kg	120 mg/kg
Bone mass and structure							
TBV, BV/TV	52.14 ± 5.29	18.95 ± 2.79 ^i^	25.73 ± 2.74 ^ik^	23.36 ± 2.47 ^i^	27.08 ± 6.49 ^i^	32.75 ± 5.50 ^ikp^	38.26 ± 8.22 ^jkp^
Tbn	36.13 ± 6.47	11.13 ± 1.36 ^i^	14.63 ± 1.60 ^ik^	14.38 ± 2.26 ^i^	15.88 ± 3.09 ^i^	22.00 ± 2.00 ^ikmo^	23.13 ± 2.53 ^ikmo^
Tbl	7.41 ± 0.74	2.34 ± 0.52 ^a^	3.28 ± 0.49 ^ad^	2.98 ± 0.19 ^a^	3.42 ± 0.68 ^ad^	4.36 ± 0.63 ^acfg^	5.23 ± 0.76 ^acef^
Tbt	124.58 ± 12.32	77.05 ± 5.96 ^a^	94.69 ± 13.44 ^a^	91.32 ± 14.36 ^a^	96.29 ± 15.15 ^a^	112.71 ± 12.97 ^dh^	115.76 ± 14.42 ^cfg^
Cbt-shaft	813.67 ± 87.66	432.65 ± 32.47 ^i^	502.08 ± 53.06 ^i^	482.92 ± 31.92 ^i^	524.97 ± 84.32 ^i^	561.55 ± 37.01 ^ikp^	686.23 ± 66.91 ^kmo^
Bone resorption							
Ocn	14.50 ± 2.20	35.63 ± 5.68 ^a^	25.00 ± 2.00 ^ac^	27.13 ± 3.40 ^ac^	24.88 ± 2.59 ^ac^	22.13 ± 2.53 ^ac^	19.88 ± 3.64 ^bcg^
OS/BS	6.49 ± 1.31	29.28 ± 3.21 ^i^	22.16 ± 1.62 ^ik^	24.29 ± 3.70 ^i^	21.38 ± 3.36 ^ik^	14.11 ± 4.38 ^jkno^	9.13 ± 1.34 ^jkmo^

Values are expressed mean ± S.D. of eight rats; ^a^ *p <* 0.01 and ^b^ *p <* 0.05 as compared with sham control by Tukey test; ^c^ *p <* 0.01 and ^d^ *p <* 0.05 as compared with OVX control by Tukey test; ^e^ *p <* 0.01 and ^f^ *p <* 0.05 as compared with RC 40 mg/kg treated rats by Tukey test; ^g^ *p <* 0.01 and ^h^ *p <* 0.05 as compared with PCP 20 mg/kg treated rats by Tukey test; ^i^ *p <* 0.01 and ^j^ *p <* 0.05 as compared with sham control by Tamhane test; ^k^ *p <* 0.01 and ^l^ *p <* 0.05 as compared with OVX control by Tamhane test; ^m^ *p <* 0.01 and ^n^ *p <* 0.05 as compared with RC 40 mg/kg treated rats by Tamhane test; ^o^ *p <* 0.01 and ^p^ *p <* 0.05 as compared with PCP 20 mg/kg treated rats by Tamhane test; OVX = Bilateral ovariectomy; PCP = Pomegranate Concentrate Powder; RC = Red clover dry extracts; L4 = fourth lumbar vertebrae; Cbt = Cortical bone thickness (Cross thickness; μm); Tbl = Trabecular bone length (Longitudinal thickness; mm); Tbn = Trabecular bone number (N/epiphyseal); Tbt = Trabecular bone thickness (Cross thickness; μm); TV/BV = Trabecular bone volume (%); OS/BS = Osteoclast cell surface/bone surface (%); Ocn = Osteoclast cell number (N/epiphyseal).

**Table 10:** Histopathology-histomorphometry for bone mass and resorption of L4 in OVX rats.

Groups	Control		References		RC:PCP 2:1 Mixture (g/g)		
	Sham	OVX	RC 40 mg/kg	PCP 20 mg/kg	30 mg/kg	60 mg/kg	120 mg/kg
Bone mass and structure							
TBV, BV/TV	56.33 ± 5.80	31.07 ± 2.17 ^a^	40.67 ± 2.21 ^ac^	38.81 ± 3.14 ^ac^	41.38 ± 4.09 ^ac^	44.97 ± 3.01 ^ach^	48.49 ± 5.58 ^aceg^
Tbn	24.50 ± 4.00	10.88 ± 2.10 ^i^	14.88 ± 1.55 ^ij^	13.38 ± 1.51 ^i^	15.25 ± 3.20 ^i^	17.25 ± 1.49 ^jko^	19.88 ± 2.17 ^kmo^
Tbl	4.45 ± 0.47	2.32 ± 0.33 ^i^	2.89 ± 0.33 ^i^	2.82 ± 0.17 ^i^	3.02 ± 0.56 ^i^	3.66 ± 0.44 ^knp^	3.89 ± 0.23 ^kmo^
Tbt	148.78 ± 14.67	109.21 ± 12.86 ^i^	125.25 ± 6.43 ^j^	124.18 ± 6.91 ^j^	128.15 ± 11.43	132.19 ± 2.27 ^l^	136.42 ± 6.01 ^kp^
Cbt-shaft	233.01 ± 32.31	143.10 ± 19.90 ^a^	176.25 ± 15.35 ^ad^	168.67 ± 9.71 ^a^	186.51 ± 30.84 ^ac^	199.67 ± 15.11 ^ac^	201.46 ± 11.24 ^ch^
Bone resorption							
Ocn	7.88 ± 1.89	22.75 ± 2.76 ^a^	18.50 ± 3.07 ^ac^	19.75 ± 1.49 ^a^	18.25 ± 2.92 ^ac^	12.38 ± 1.30 ^aceg^	10.75 ± 1.67 ^ceg^
OS/BS	5.75 ± 0.93	20.40 ± 2.67 ^a^	16.03 ± 2.06 ^ac^	17.13 ± 1.55 ^ad^	15.14 ± 2.96 ^ac^	9.35 ± 1.26 ^bceg^	7.61 ± 1.54 ^ceg^

Values are expressed mean ± S.D. of eight rats. ^a^ *p <* 0.01 and ^b^ *p <* 0.05 as compared with sham control by Tukey test; ^c^ *p <* 0.01 and ^d^ *p <* 0.05 as compared with OVX control by Tukey test; ^e^ *p <* 0.01 and ^f^ *p <* 0.05 as compared with RC 40 mg/kg treated rats by Tukey test; ^g^ *p <* 0.01 and ^h^ *p <* 0.05 as compared with PCP 20 mg/kg treated rats by Tukey test; ^i^ *p <* 0.01 and ^j^ *p <* 0.05 as compared with sham control by Tamhane test; ^k^ *p <* 0.01 and ^l^ *p <* 0.05 as compared with OVX control by Tamhane test; ^m^ *p <* 0.01 and ^n^ *p <* 0.05 as compared with RC 40 mg/kg treated rats by Tamhane test; ^o^ *p <* 0.01 and ^p^ *p <* 0.05 as compared with PCP 20 mg/kg treated rats by Tamhane test; OVX = Bilateral ovariectomy; PCP = Pomegranate Concentrate Powder; RC = Red clover dry extracts; L4 = fourth lumbar vertebrae; Cbt = Cortical bone thickness (Cross thickness; μm); Tbl = Trabecular bone length (Longitudinal thickness; mm); Tbn = Trabecular bone number (N/epiphyseal); Tbt = Trabecular bone thickness (Cross thickness; μm); TV/BV = Trabecular bone volume (%); OS/BS = Osteoclast cell surface/bone surface (%); Ocn = Osteoclast cell number (N/epiphyseal).

## 4. Discussion

Hormone therapy is often used to relieve climacteric symptoms; however, serious concerns have been raised regarding the use and safety of hormone replacement therapy over long treatment periods in relation to cardiovascular events and breast cancer [20,21,22]. Consequently, many researchers have sought alternative therapies, including the use of PEs to relieve menopausal symptoms [23,24,25]. Therefore, our study is meaningful because the RC:PCP 2:1 mixture we applied potently reduced climacteric symptoms (*i.e.*, estrogenic, anti-osteoporotic, and anti-obesity effects).

First, we observed the anti-obesity effects in OVX rats; the 120 mg/kg mixtures reduced food and water consumption compared with a single formula of RC and PCP. In addition, the 120 mg/kg mixtures caused increases in fecal and urinary excretion compared with RC or PCP treatment but did not influence food and water intake significantly. These results indicate that the addition of appropriate amounts of PCP increased the inhibitory effect in OVX-induced estrogen deficiency and related obesity. These effects are attributed to the enhancement of digestive tract motility or diuretic effects, along with estrogenic inhibition by increasing the satiating potency of digestive hormones, cholecystokinin (CCK) [26], and glucagon [27]. Increased digestive motility also induced increased fecal excretion and consequently induced decreased the body weight of rats [28], similar to the decreased body weight induced by diuretics in rats [29]. Estradiol is involved in the regulation of food consumption and body weight mainly by modulating the potency of feedback signals that control meal size [30]. The most investigated mechanism indicates that estradiol increases the satiating potency of exogenous and endogenous CCK [26]. A similar explanation may be applicable for glucagon because the effects of glucagon and glucagon antibodies on meal size control are amplified by estradiol in OVX rats [27]. The absence of estradiol leads to a temporary increase in food consumption and a sustained increase in body weight [31]. This phenomenon is of clinical relevance because estradiol levels decrease in postmenopausal females; importantly, postmenopausal females constitute a high percentage of the obese population [32]. Estrogen depletion caused by ovariectomy in rats induced severe increases in food and water intake, but not urinary and fecal excretion, and it facilitated body fat deposition, especially in the abdominal cavity as stated by other investigators [32,33]. The accumulation of fat or increase in fat deposition in the body is a major characteristic of obesity, and cellular hypertrophy appears to be the major mode of expansion of intra-abdominal adipose tissue in rodents [18].

Next, we found that the 120 mg/kg mixtures exhibited stronger inhibitory effects against ovariectomy-induced estradiol depletion and related uterine atrophic changes compared with a single formula of PCP. RC and PCP and their combinations are considered to exert protective effects against ovariectomy-induced uterine atrophy. The increase in uterine mass is mainly attributed to uterine water inhibition and/or cell proliferation [34] and is mediated through estrogen receptor (ER)-α [35]. PEs can mimic estrogen [6] in biological systems due to their structural similarity to estrogen. PEs and isoflavonoids can have a weak estrogen-like effect by binding to both ER-α and -β in various tissues [35]. Estrogen deficiency is accompanied by marked atrophy of organs such as the uterus and vagina [33].

Lastly, we determined the anti-osteoporotic effect using various data, including BMD, bALP, and trabecular and cortical bones. As a result, estrogen-deficient osteoporosis was significantly inhibited in RC, PCP, and mixture-treated OVX rats. Specifically, the 120 mg/kg mixtures showed a stronger inhibitory effect against estrogen-deficient osteoporosis compared with a single formula of RC and PCP. Osteoporosis, a metabolic bone disease, results from a disturbance in normal bone remodeling, changing the balance to bone resorption over formation. Osteoporosis is caused by an imbalance between bone resorption and bone formation and results in bone loss and fractures after mineral flux [36]. Estrogen-deficient OVX osteoporosis animal models have been used to evaluate osteoporotic drugs [37]. Serum osteocalcin levels are generally considered to be a marker of bone turnover and serum bALP levels to be a marker of bone formation [38]. As progression of OVX related osteoporosis, serum osteocalcin levels were generally increased along the increases of bone turn over, but serum bALP contents were decreased along inhibition of bone formations [38,39]. On the contrary, the serum osteocalcin levels were decreased, but serum bALP levels were increased in present study, which suggested that the mixture treatment activated the osteoblast differentiation and inhibited the bone mineralization and turn over [40]. BMD provides information regarding the efficacy of anti-osteoporotic agents [41]. Microscopic observations of bones also provide valuable information regarding bone morphology [42]. In osteoporotic animals, the histological profiles are clearly changed compared with sham controls regardless of the cause, especially in the trabecular and cortical bones. The efficacy of various anti-osteoporotic agents has been evaluated using bone histology [43]. Namely, some histomorphometrical indices for bone mass and bone formation are decreased concomitantly with increased bone resorption, and this data can help predict the efficacy of anti-osteoporotic agents [43].

Many studies have shown that pomegranate juice and pomegranate polyphenol extracts can prevent many types of cancer, cardiovascular disease, diabetes, Alzheimer’s disease, arthritis, and colitis [8,44,45]. Recent reports have shown that pomegranate seed oil and pomegranate juice contain several species of flavonoids and anthocyanidins, and that their antioxidant activity is three times more potent than that in red wine or green tea extract [9,10]. Recently, RC botanical dietary supplements have received significant attention for their potential use in the treatment of menopausal symptoms. The estrogenic activity of RC is mainly due to isoflavones and, to a lesser extent, coumestans [4]. Isoflavones have various biological activities and can improve metabolic symptoms [46] and bone-protective effects [47] in menopause. Isoflavonoids exert a weak estrogen-like effect by binding to ER-α and -β in various tissues [6,35,48]. Furthermore, flavonoids have the ability to interact with estrogen receptors and to control the activity of CYP19, an important enzyme in estrogen biosynthesis, and/or steroid dehydrogenases, (e.g., 11β-hydroxysteroid dehydrogenase) [49]. These effects induce various alterations causing a change in the overall hormonal balance, resulting in protection against bone loss and reducing osteoporotic effects and other menopausal symptoms [50]. Conversely, estrogen-mediated repression of phase 2 enzyme activities may increase cellular oxidative DNA damage that can result ultimately in the formation of cancer [51]. This DNA damage is inhibited by superoxide dismutase 3 induced by antioxidants [52]. These findings indicate that the beneficial effects of RC [3,4,25] and PCP [10,11] on postmenopausal symptoms are mainly due to the phytoestrogenic effects of isoflavonoids and related antioxidant pathways [12,13]. Therefore, appropriate mixtures consisting of RC and PCP may show greater favorable anti-climacteric effects by combination of isoflavonoids and ellagic acid [53,54]. The RC and PCP mixture may enhance direct free radical scavenging and indirectly induce antioxidative enzymes.

Our results show that RC:PCP 2:1 mixture relieves climacteric symptoms through anti-osteoporotic and anti-obesity activities, but, there are some limitations in this study. The estrogen-deficient OVX model is useful for evaluating anti-climacteric effects. Since several symptoms of climacterium are clearly induced by ovariectomy within 4 to 6 weeks postoperatively, the rat model was chosen to investigate the mechanisms responsible for menopause-related complications in humans due to many similarities with postmenopausal climacterium symptoms in rats. However, menopause is related with aging and postmenopausal symptoms occurring in elderly females. A limitation of this study is that the anti-climacterium effects were evaluated in young and not older female rats; the effects of RC and PCP against climacterium may be different in older animal models. Anticlimacterium effects of RC:PCP mixture were assumed to be related with the major active components of RC and PCP, however, their principal phytochemical constituents weren’t measured in plasma or the respective tissues. In addition, characterization of the phenolic composition of RC and PCP weren’t fully analyzed; only 4 types of isoflavone (genestin, biochain A, formononetin, and daidzein) were clarified in RC and ellagic acid was measured in PCP. Thus, these effects of RC:PCP mixture weren’t discussed precisely. To clearly confirm the anticlimaterium effects of RC:PCP 2:1 mixture, further studies should analyze characterization of the full phenolic composition of each material and evaluate their principal phytochemical constituents in plasma or the respective tissues.

## 5. Conclusions

Collectively, our results suggest that a potential effect of RC-PCP mixture increased the anti-climacterium, anti-obesity, and anti-osteoporotic effects of RC in OVX rats. Therefore we suggest that the RC:PCP 2:1 mixture is a promising new potent protective agent for relieving climacterium symptoms, especially, obesity, and osteoporosis, in menopausal females.

## Supplementary Files

Supplementary File 1
