# Supplementary material for: Dried Pomegranate Potentiates Anti-Osteoporotic and Anti-Obesity Activities of Red Clover Dry Extracts in Ovariectomized Rats
Source: Nutrients. 2015 Apr 9;7(4):2622–47. doi: 10.3390/nu7042622 (PMC4425164; doi:10.3390/nu7042622)
Supplement: Supplementary File 1 [file nutrients-07-02622-s001.pdf]

evear

extraction solutions

certificat of analysis

Red clover dry extract 8% isoflavones by HPLC

Batch n°: E312081
Botanical name: Trifolium pratense L.
Used part: aerial parts
Package: 25/50 kg drum
Manufacturing date: 03/2013
Expiry date: 03/2016

Storage: closed container in cold and dry area away from light.

	ANALYSES	SPECIFICATIONS	RESULTS	METHODS
CHARACTERISTICS	Organoleptic	Brown Greenish fine powder with characteristic odour and taste	CONFORM	Euro Pharma 2.3.4
TRIAL	Loss on drying m/m in per cent<br>Heavy metal<br>Granulometry	< 6.0<br>< 20 ppm<br>97% pass through 300 µm	CONFORM<br>CONFORM<br>CONFORM	Euro Pharma 2.8.17<br>Euro Pharma 2.4.8 C<br>Euro Pharma 2.9.12
DOSAGE	Isoflavones content<br>(Biochanin A + genistein)/(Dadzein +formonetin)<br>(Genistein + Daidzein)/(Total isoflavones G+D+B+F)	**>8%**<br>**0,1-10**<br>**<10**	**9,1%**<br>**CONFORM**<br>**CONFORM**	HPLC
MICRO BIOLOGICAL CHARACTERS	Total aerobes germs at 30°C<br>Yeast and moister<br>Enterobacteries and others bacteria gram-negatives<br>Staphylococcus aureus<br>Escherichia coli<br>Salmonella	< 10 000 germs/g<br>< 100 germs/g<br>< 100 germs/g<br>None in 1 g<br>None in 1 g<br>None in 10 g	CONFORM<br>CONFORM<br>CONFORM<br>CONFORM<br>CONFORM<br>CONFORM	Euro Pharma 2.6.12 / 2.6.13 or AFNOR

evear Extraction Végétale et Aromatique
Félines — F — 49320 COUTURES
Tél : 00 33 2 41 57 68 81
Fax : 00 33 2 41 57 68 79
info@evear-extraction.com
www.evear-extraction.com

제 D2013060438 호

검 사 성 적 서 Test Report

검체명	Red Clover		제조일자<br>(유통기한)	
의뢰인	업체명	(주)건강사랑	성 명	이해연
	주소	경기 화성시 봉담읍 분천리 96-11,114-2		
제조번호		Betch-A 2312081	접수년월일	2013-06-13
검사의뢰목적		참고용	검체접수번호	D2013060438

귀하가 우리 연구원에 검사의뢰한 결과는 다음과 같습니다.

검사관련 총 책임자:김 천 회

시험항목	결과	검사담당자
Genistein(%)	0.62%	강동희
Biochanin A(%)	5.43%	강동희
Formononetin(%)	3.66%	강동희
Daidzein(%)	0.47%	강동희

분석방법-업체제공.

2013 년 6 월 27 일

한 국 기 능 식 품 연 구 원 장

(사)한국건강기능식품협회 부설 한국기능식품연구원 http://www.khsi.re.kr 전화번호(031)628-2400 FAX(031)628-0400~1

ASYA MEYVE SUYU VE GIDA SANAYİ A.Ş.	ANALYSIS CERTIFICATE	Doküman No :F108<br>İlk Yayın Tarihi :<br>Revizyon No :'00<br>Revizyon Tarihi :-

PRODUCT NAME: Dried Pomegranate Concentrate Powder

PRODUCTION DATE: 2013-04-17

EXPIRY DATE 2014-04-17

BATCH NO: 11NT10

PHYSICAL & CHEMICAL ANALYSIS RESULTS

**TASTE-AROMA**	Natural pomegranate juice taste and aroma
**MOISTURE**	<5%
**CHEMICAL SUBSTANCE**	None
**PRESERVATIVES**	None

MICROBIOLOGICAL ANALYSIS RESULTS

**TOTAL COUNT (cfu/ml)**	<1000
**MOULD (cfu/ml)**	<100
**YEAST (cfu/ml)**	<100
**COLIFORM (cfu/ml)**	None
**E. COLI (cfu/ml)**	None
**HEAT RESİSTANT MOLD(cfu/ml)**	Negative

QUALTY ASSURANCE LEADER

제 D2013050939 호

검 사 성 적 서 Test Report

검체명	pomegranate powder 10	제조일자<br>(유통기한)	
의뢰인 업체명	(주)건강사랑	성 명	이해연
의뢰인 주소	경기 화성시 봉담읍 분천리 96-11,114-2		
제조번호	11NT10	접수년월일	2013-05-23
검사의뢰목적	참고용	검체접수번호	D2013050939

귀하가 우리 연구원에 검사의뢰한 결과는 다음과 같습니다.

검사관련 총 책임자:김 천 회

시험항목	결과	검사담당자
엘라그산(mg/g) Ellagic acid (mg/g)	0.90mg/g	유재명

분석법-업체제공

2013 년 5 월 28 일

한 국 기 능 식 품 연 구 원 장

(사)한국건강기능식품협회 부설 한국기능식품연구원 http://www.khsi.re.kr 전화번호[illegible] FAX(031)628-0400~1
